# Assessing Weather Services for Rural Fishing and Farming Communities in Uganda

**DOI:** 10.4236/ajcc.2020.92011

**Published:** 2020-06-19

**Authors:** Patrick Kibaya, Ian G. Munabi, Frank Annor, John Baptist Kaddu

**Affiliations:** 1 Climate Change Adaptation Innovation (CHAI), Kampala, Uganda; 2 Department of Anatomy, School of Biomedical Sciences, Makerere University College of Health Science, Kampala, Uganda; 3 Department of Water Resources, Faculty of Civil Engineering and Geosciences, Delft University of Technology, Delft, The Netherlands; 4 Civil Engineering Department, Kwame Nkrumah University of Science and Technology, Kumasi, Ghana; 5 Department of Zoology, Entomology and Fisheries Sciences, School of Biosciences, Makerere University College of Natural Sciences, Kampala, Uganda

**Keywords:** Climate Change, Resilience, Early Warning Systems, Gender, Africa

## Abstract

Climate-related hazards like drought are associated with loss of life and lead to food insecurity in many parts of sub-Saharan Africa. Food insecurity, which affects more than 220 million sub-Saharan Africans, manifests as starvation that leads to more than 50% of children under the age of 5-years presenting as underweight for age in many communities on the continent. This household survey reports the means by which rural fisher folk and farming communities in Uganda gained access to early warning meteorological information. The survey covered five districts across different climatic zones in Uganda and recruited a total of 405 respondents with an average age of 41 years (SD 16). Economic activity was used to categorize each of the five districts into farming (crops and livestock) and fishing areas. The results showed that most respondents were unaware of drought as one of the climate-related hazards. Compared to respondents from the fishing communities, the respondents from farming communities were more likely to be receiving weather-related information (*P*-value < 0.01). There were 204/405 (50.37%) female respondents who, compared to male respondents, were less likely to have access to weather information, less willing to pay for weather information, and less likely to have and or own devices like a radio for receiving weather information. The survey demonstrated that: 1) there were gaps in the knowledge about climate-related hazards, 2) there is a need for additional interventions targeting fisher folk communities access timely weather information, and 3) introducing user paid access to weather information may increase climate-related gender-based disparities.

## 1. Introduction

Climate-related hazards like drought are known to cause loss of life and livelihood of communities that depend on rain-fed agriculture (Akwango, Obaa, Turyahabwe, Baguma, & Egeru, 2017; Shiferaw et al., 2014). This is especially true for sub-Saharan Africa (SSA) where drought is identified as one of the leading causes of food insecurity (Shiferaw et al., 2014). This food insecurity as a result of climate change, affects more than 220 million sub-Saharan Africans and leads to: increase international migration out of sub-Saharan Africa (Sadiddin, Cattaneo, Cirillo, & Miller, 2019), increased civil strife or wars as people move to or struggle to control places with an abundance of food (Ujunwa, 2019), and in Uganda additionally manifests as more than 50% of children under the age of 5-years presenting as underweight for age.

In Uganda individuals who are involved in agriculture and constitute 80% of the labour force include: smallholder farmers, fishermen, and pastoralists. Studies show that Early Warning Systems (EWS) save lives and reduce economic losses from floods, droughts, storms, and other weather-related hazards (Akwango et al., 2017). The challenge remains that farmers, fishermen and community support institutions in Uganda receive little or no relevant information to help them cope with droughts, floods and other climatic stresses. This study set out to determine the means by which rural fisher folk and farming communities in Uganda gained access to early warning meteorological information.

## 2. Methods

In this cross sectional survey, eight districts from various parts of Uganda were purposively sampled to represent different climatological and agro-ecological zones of Uganda. Each of the eight-study districts was divided into farming (crops and livestock) and fishing areas using the known most common economic activity for each area. The target study sample size of 577 household heads was obtained using the following assumptions: potential target population of 2,187,500 house hold heads in Uganda; 50% of these households are affected in some form or way by climate change; for *α* = 95%; power of 0.95; a design effect of 1.5, using the online sample size calculator for sample size for a proportions found at http://www.openepi.com/.

### 2.1. Participant Selection

For each area in the selected district pairs of villages were purposively selected from different counties to give a total of 4 villages per district. From each study village, 20 households were selected using a stratified systematic random sampling strategy. The selection of the participating villages in the identified 8 districts was done purposively to ensure that 1) 12% of the selected villages were predominantly composed of fisher folk; 2) that the selected villages would represent all the 14 climatic zones of Uganda; 3) each of the selected villages was within a 40 Kilometer radius of the nearest Weather Station; and 4) that at least a quarter (160) of the respondents were female. Identified household heads who were selected and those that failed or refused to participate in the study were excluded.

### 2.2. Study Tool and Analysis

A household survey tool was designed to capture information on the following: respondent identifier information; level of education; means of livelihood; climate-related hazards and impacts; technology access and willingness to pay for early weather warning information. The data from the various households was captured using open data kit digitized questionnaires on hand-held devices that transmitted the collected information in real time after a series of validation checks to a data capture server at the end of each interview. The information from the data capture server was downloaded for analysis using STATA 13 to generate both the descriptive and inferential statistics. The level of significance for the tests was set at 0.05.

## 3. Results

Due to logistical reasons it was only five of the previously identified eight districts were sampled. A total of 405 respondents were recruited, which reduced the power of the survey from 0.95 to 0.90. The respondents were from the following districts of Uganda: 97/405 (23.95%) from Jinja in the East; 89/405 (21.98%) from Kabaale in the South West; 81/405 (20.00%) from Kalangala one of the island districts in Uganda’s Lake Victoria; 57/405 (14.07%) from Nakasongola in Central Uganda in the cattle corridor and 81/405 (20.00%) from Nebbi district from north western part of Uganda. The average age of the survey respondents was 41 years (SD 16). There were more female respondents (204/405, 50.37%) than male respondents (200/405, 49.38%). The respondents were: 172/405 (42.47%) respondents who were husbands; 161/405 (39.75%) wives; 55/405 (13.58%) children; and 16/405 (3.20%) other respondents who included relatives to the surveyed households. Table 1 provides a summary of the different levels of education for the various respondents in the survey. In this Table 1, note, that none of the respondents had A-level or University level training. The majority of respondents were married (239/405, 59.16%), followed by 63/405 (15.59%) who were co-habiting, 54/405 (13.37%) who were single with the rest 48/405 (11.88%) living as either divorced, separated or widowed. The mean household size was 4 individuals with a range of 1 to 9 individuals.

### 3.1. Livelihood

The majority of respondents resided in villages that were described as either a farming community (296/404, 73.27%) or a fishing community (108/404, 26.73%) depending on the most common form of occupation in the community. Farming was identified as the main source of income for the majority of the respondents, (250/403, 62.03%). There were also 77/403 (19.11) respondents involved in fishing, 20/403 (4.96%) respondents involved in livestock rearing, 18/403 (4.47%) wage or salary earners, with the remaining 38/403 reporting other main sources of income that included remittances, milk and forest related products. There were 393 respondent households with a mean 5.20 (SD 18.63) acres of land under cultivation. Respondents from farming communities had larger acreage under cultivation (mean 6.52 acres, SD 21.28) compared with those from fishing communities (mean 1.18 acres, SD 0.22). This was significant (*P*-value = 0.01, T score = 2.46). On average 4.38 acres (SD 16.29) of land was under rain fed cultivation compared to 0.12 acres (SD 0.52) under irrigated cultivation. The fisher folk on the other hand reported catching and selling fish equivalent to 23.51 kilograms (SD 11.65) of fish per month.

### 3.2. Exposure to Climate Hazards

The majority of respondents (398/405, 98.27%) did not have lightning conductors at their residences. Table 2 provides a summary of the other climate-related hazards that were reported to have affected respondents in the last 12 months. It is important to note that this table only has those items identified by respondents as having affected them during the survey. The other hazards that were included in the data collection tool and not mentioned by the respondents were: drought, prolonged dry spell, floods, unpredictable rainfall pattern, hailstorms, thunderstorms, lightning, pests affecting crops and increase in plant diseases. In comparison to the fisher folk communities, respondents from farming communities were less likely to mention any of the identified climatic hazards in Table 2. This was not significant (Odds ratio 0.65, 95% CI 0.39 to 1.07, *P*-value 0.09). There was also no significant difference in odds of exposure to the reported climate-related hazards with respect to the amount of cultivated land the household reportedly had (Odds ratio 1, 95% CI 0.98 to 1.01 *P*-value = 0.49).

### 3.3. Access to Weather Information

The majority of the respondents (223/403, 55.33%) were receiving weather related information. Respondents from farming communities were more likely to be receiving weather related information compared to those from fishing communities. This was significant (Odds ratio 2.48, 95% CI 1.58 to 3.90 *P*-value < 0.01). With respect to gender 111/199 (55.78%) male respondents reported having received some form of weather information compared with 111/203 (54.68%) female respondents. The above differences in responses with respect to gender were not significant (Odds ratio 1.00, 95% CI 0.68 to 1.47, *P*-value = 1). There was no significant difference in access to weather information with respect to the overall respondent’s level of education, on sub analysis we observed that: respondents with no formal education (Odds ratio 5.13 95% CI 1.68 to 15.64 *P*-value < 0.01); respondents with 0-level education (Odds ratio 7.87 95% CI 2.53 to 24.50 *P*-value < 0.01); and respondents with college certificates (Odds ratio 5.28 95% CI 1.76 to 15.87 *P*-value < 0.01), were significantly more likely to access weather information than those with religious education only. Table 3 provides a summary of the various types of weather information available to the respondents. In this table note that information on seasonal (3 months) weather forecasts, followed by the daily weather forecasts were the most highly ranked forms of weather information identified by respondents. The respondents also identified various sources of weather information available to them.

### 3.4. Perceived Accuracy of Weather Information

The majority of the respondents (176/397, 44.33%) did not know about the quality of weather information they were receiving. In comparison 139/397 (35.01%) respondents thought that the information was somewhat accurate; 46/397 (11.59%) thought it was not accurate and 36/397 (9.07%) thought the information they were receiving was accurate. Additional analysis showed that there was no significant difference in responses on the accuracy of weather information with respect to gender (Odds ratio 1.19 95% CI 1.00 to 1.44, *P*-value 0.05) or the respondent’s level of education (regression coefficient 0.04 95% CI −0.09 to 0.02 *P*-value = 0.16). Table 4 provides a summary of the information sources identified by the respondents. In this table note that the FM radio stations were the most frequently identified sources of information for respondents.

### 3.5. Willingness to Pay for Weather Information

Only 193/403 (47.89%) of the respondents indicated that they were willing to pay for weather information compared with 210/403 (52.11%) who indicated that they were not willing to pay for weather information. Compared with male respondents (114/199, 57.29%) who were willing to pay for weather information, Female respondents (78/203, 38.42%) were less likely to pay for weather related information. This gender related difference in willingness to pay was significant (Odds ratio 0.47 95% CI 0.31 to 0.70 *P*-value < 0.01). Also, respondents from predominantly farming communities were less likely to indicate a willingness to pay for weather information compared to predominantly fishing communities. This was not significant (Odds ratio 0.72 95% CI 0.46 to 1.21 *P*-value = 0.15). When asked how much they would be willing to pay on a weekly basis for weather information, 18/210 (8.57%) said they could pay less than 500 UGX, 37/210 (17.62%) were willing to pay between 500 UGX to 1000 UGX and the majority 155/210 (73.81%) were willing to pay more than 1000 UGX per week for weather information. Note that 1000 UGX was approximately equivalent to 0.25 USD at the time of the survey.

### 3.6. Devices Used to Access Weather Information

Table 5 provides a summary of the devices that are: owned, easiest to use and frequently used by different respondents. In this table it is important to note that the ordinary mobile phone and radio were the most frequently owned and used devices in the study population. There was no significant difference in responses to the questions on use, frequency of use or ease of device use with respect to gender or predominant occupation of the study community. It was observed that there were no significant differences in responses to the questions on: device ownership (Odds ratio 1.10 95% CI 0.88 to 1.36 *P*-value = 0.40); frequent use of the device (Odds ratio 1.00 95% CI 0.87 to 1.16 *P*-value = 0.94); and simplicity of use (Odds ratio 1.00 95% CI 0.87 to 1.16 *P*-value 0.95), of the various identified devices in Table 5 with respect to gender. Similar sets of observations were made with respect to the respondent’s level of education and: device ownership (Odds ratio 0.83 95% CI 0.68 to 1.01 *P*-value = 0.06); frequent use of devices (Odds ratio 1.01 95% CI 0.89 to 1.16 *P*-value = 0.81); and simplicity of device use (Odds ratio 0.92 95% CI 0.81 to 1.04 *P*-value = 0.20).

## 4. Discussion

The study set out to determine the means by which rural fisher folk and farming communities in Uganda gained access to early warning meteorological information. It was noted that 55.33% of the survey respondents were receiving weather information predominantly from the FM radio stations despite the high prevalence of mobile phones in the country. The weather information received was mainly in the form of the three-monthly advance notices of weather conditions. It is of interest to note that individuals in farming communities were twice as likely to receive weather information compared to people in fishing communities (*P*-value < 0.01). Elsewhere it has been noted that the nature and occupation of the fisher folk may leave them less psychologically aware of how closely climate change affects them as individuals and as a community compared to the farmers (Sinha & Das, 2019; Steynor & Pasquini, 2019). This may explain the increased exposure to risks associated with climate change for the fisher folk.

The other factor that may affect the use of EWS notifications is the accuracy of the information sent to the users. From this survey most of the respondents noted that the information they received was not accurate. Here we think that accurate refers to weather information being local enough for the respondents to use in their day-to-day decisions as has been observed in travel choices made by tourists (Scott & Lemieux, 2010). The tougher economic conditions of life in the fisher folk communities and questionable accuracy of the information received may have a role to play in observed differences in weather information use by this community compared to the farmers (Sinha & Das, 2019). On the other hand, the difference in access to weather information may also point to perceived differences in value attached to weather information by the two communities, with the farming communities being more dependent on rainfed forms of livelihood. A possible solution to this would be to tailor the early warning information to suit the needs of the fisher folk community by for example giving hourly notices as opposed to week or season long information (Singh et al., 2018; Steynor & Pasquini, 2019).

The economic/financial value of weather information to the respondents is demonstrated by the responses to their willingness to pay for weather information, with the majority willing to pay more than 1000 UGX (25 US cents) per week. Note that the female respondents were less likely than male respondents to indicate a willingness to pay for weather related information. This has been documented by other research papers from eastern Uganda and other parts of Africa (Dah-gbeto & Villamor, 2016; Nabikolo, Bashaasha, Mangheni, & Majaliwa, 2012). It is important to cautiously deconstruct this gender unwillingness to pay for access to weather information as advised by Rao et al. (2019). Deconstructing should thus involve a detailed exploration of women’s roles in the generation and control of economic/financial value in the home as defined by their communities or culture (Eastin, 2018; Hyland & Russ, 2019; Rao et al., 2019; Yadav & Lal, 2018). Without this deconstruction any form of paid for access to weather information may aggravate the gender differences in the observed response to climate change events for this population.

The observed low use of lightning conductors for 98.27% of homesteads visited points to a very low level of awareness about the various extreme weather events. Also, as summarized in Table 4, the respondents identified only a small number of the extreme weather events for both communities. This points to a potential knowledge gap on the part of the respondents about the nature of extreme weather events associated with climate change and how this can affect them. This lack of knowledge of extreme weather events or climate change is recognized as one of the barriers to climate change adaptation response (Moser & Ekstrom, 2010). The respondent’s failure to identify some of the more common extreme weather events known to affect the region even as most of them continue receiving weather related information is an additional concern with regards to the resilience of this population (van der Keur et al., 2016). This lack of knowledge about extreme weather events may affect climate change adaptation response at the individual, community and the local leadership levels of society (Keim, 2008).

### Limitations

The study experienced logistical challenges that reduced the representativeness of the study with respect to the various climatic zones in the country. This led to a reduction in the study sample size and a minor reduction in the power of the study from 0.95 to 0.90. The other limitation is that the study population was predominantly rural given the focus on fisher folk and farming communities, leaving out residents in urban communities. These limitations point to a need for cautious extrapolation of the study conclusions to other communities in Uganda.

## 5. Conclusion

The survey demonstrated that there were gaps in the respondent’s knowledge about climate-related hazards. The respondents, mainly farmers and fisher folks were interested mostly in the impact of the weather and climate on their direct activities and not even on the extreme climate change effects such as drought and floods in the community at large. Issues such as food insecurity which leads to underweight in children especially those under five were not well recognized by the respondents. Farmers seemed more comfortable with the use of climate information (a more general seasonal forecast) compared to fishermen. There is a need for additional targeted interventions to ensure that the fisher folk communities’ access timely localized weather information relevant to their activities. Introducing user “paid for access” to weather information may lead to increased climate change gender-based disparities in this population. While mobile phone penetration has improved sharply in Uganda (>60%) and other parts of Africa (>80%), there is a preference for mass media (such as FM radio) as the source for weather information. The difference in access to weather information from farmers and fisher folks, may also point to perceived differences in value attached to the weather information by them.

## Figures and Tables

**Table 1.** Summary of the respondent’s levels of education.

Level of education	Male (%)	Female (%)	Total (%)

Religious education only	1 (100)	0 (0)	1 (0.25)
No formal education	36 (41.83)	51 (58.62)	87 (21.48)
Lower primary (P1 to P4)	11 (35.48)	20 (64.52)	31 (7.65)
Upper primary (P5 to P7)	45 (52.33)	41 (47.67)	86 (21.23)
Ordinary level (Senior 1 to 4)	42 (52.50)	38 (47.50)	80 (19.75)
Advanced level (senior 5 to 6)	0	0	0
College certificate	50 (49.50)	51 (50.50)	101 (24.94)
College diploma	15 (78.95)	4 (21.05)	19 (4.69)
University degree	0	0	0
Total	200 (49.38)	205 (50.62)	405 (100)

**Table 2.** Climatic hazards affecting respondents.

Hazards	Did the hazard affect the respondent		
	Yes	No	Total

Pests affecting livestock	96	239	335
Climate-related human diseases	88	253	341
Termites	104	230	334
Others	54	280	334
Total	342	1002	1344

**Table 3.** Ranking of types of weather information.

Type of information	Ranking of information				

Rank	1	2	3	4	Total

Daily weather forecasts	61	21	7	14	103
10-day weather forecast	5	2	5	6	18
Seasonal (3 months) forecast	81	28	10	8	127
Early warning on extreme rains	39	29	21	17	106
Early warning on extreme heat	7	9	14	17	47
Early warning on drought	39	29	18	23	109
Early warning on thunderstorms	10	3	3	1	17
Early warning on destructive winds	1	2	1	4	8
Early warning on mudslides	8	1	11	7	27
Others	5	1	3	5	14
Total	256	125	93	102	576

**Table 4.** Sources of weather information.

Type of information	Source of information						

Source	1	2	3	4	5	6	Total

Daily weather forecasts	0	1	68	6	0	0	75
10-day weather forecast	1	0	11	1	0	0	13
Seasonal (3 months) forecast	2	0	97	6	1	1	107
Early warning on extreme rains	1	0	70	7	0	1	79
Early warning on extreme heat	2	0	38	3	0	0	43
Early warning on drought	1	0	81	5	0	0	87
Early warning on thunderstorms	0	0	10	3	0	0	13
Early warning on destructive winds	0	0	4	0	0	0	4
Early warning on mudslides	1	0	18	3	1	0	23
Others	0	0	9	2	0	0	11
Total	8	1	406	36	2	2	455

Key: 1-mobile phone (text message), 2-Moblie phone (voice), 3-Local FM radio, 4-Television, 5-community radio, 6-Community extension workers

**Table 5.** Devices used to access weather information.

Device	Numbers that		
	Owned	Use often	Found simple to use

Ordinary mobile phone	68	181	186
Smart phone	2	6	6
Television	3	13	7
Radio	77	140	159
Total	150	340	358

## List of Abbreviations

EWS: Early warning systems
FIC: Fogarty International Center
FM: Frequency Modulation
NHLBI: National Heart, Lung, and Blood Institute
NIDCR: National Institute of Dental & Craniofacial Research
NIMHD: National Institute on Minority Health and Health Disparities
NINDS: National Institute of Neurological Disorders and Stroke
OD: Office of the Director, National Institutes of Health
SD: Standard deviation
SSA: Sub-Saharan Africa
UGX: Uganda Shillings
UNCST: Uganda National Council of Science and Technology
US: United States of America

## References

[R1] Akwango D, Obaa BB, Turyahabwe N, Baguma Y, & Egeru A. (2017). Effect of Drought Early Warning System on Household Food Security in Karamoja Subregion, Uganda. Agriculture & Food Security, 6, Article No.: 43. 10.1186/s40066-017-0120-x

[R2] Dah-gbeto AP, & Villamor GB (2016). Gender-Specific Responses to Climate Variability in a Semi-Arid Ecosystem in Northern Benin. Ambio, 45, 297–308. 10.1007/s13280-016-0830-5 27878534 PMC5120022

[R3] Eastin J. (2018). Climate Change and Gender Equality in Developing States. World Development, 107, 289–305. 10.1016/j.worlddev.2018.02.021

[R4] Hyland M, & Russ J. (2019). Water as Destiny—The Long-Term Impacts of Drought in Sub-Saharan Africa. World Development, 115, 30–45. 10.1016/j.worlddev.2018.11.002

[R5] Keim ME (2008). Building Human Resilience: The Role of Public Health Preparedness and Response as an Adaptation to Climate Change. American Journal of Preventive Medicine, 35, 508–516. 10.1016/j.amepre.2008.08.022 18929977

[R6] Moser SC, & Ekstrom JA (2010). A Framework to Diagnose Barriers to Climate Change Adaptation. Proceedings of the National Academy of Sciences of the United States of America, 107, 22026–22031. 10.1073/pnas.1007887107 21135232 PMC3009757

[R7] Nabikolo D, Bashaasha B, Mangheni MN, & Majaliwa JGM (2012). Determinants of Climate Change Adaptation among Male and Female Headed Farm Households in Eastern Uganda. African Crop Science Journal, 20, 203–212.

[R8] Rao N, Lawson ET, Raditloaneng WN, Solomon D, & Angula MN (2019). Gendered Vulnerabilities to Climate Change: Insights from the Semi-Arid Regions of Africa and Asia. Climate and Development, 11, 14–26. 10.1080/17565529.2017.1372266

[R9] Sadiddin A, Cattaneo A, Cirillo M, & Miller M. (2019). Food Insecurity as a Determinant of International Migration: Evidence from Sub-Saharan Africa. Food Security, 11, 515–530. 10.1007/s12571-019-00927-w

[R10] Scott D, & Lemieux C. (2010). Weather and Climate Information for Tourism. Procedia Environmental Sciences, 1, 146–183. 10.1016/j.proenv.2010.09.011

[R11] Shiferaw B, Tesfaye K, Kassie M, Abate T, Prasanna BM, & Menkir A. (2014). Managing Vulnerability to Drought and Enhancing Livelihood Resilience in Sub-Saharan Africa: Technological, Institutional and Policy Options. Weather and Climate Extremes, 3, 67–79. 10.1016/j.wace.2014.04.004

[R12] Singh C, Daron J, Bazaz A, Ziervogel G, Spear D, Krishnaswamy J, (2018). The Utility of Weather and Climate Information for Adaptation Decision-Making: Current Uses and Future Prospects in Africa and India. Climate and Development, 10, 389–405. 10.1080/17565529.2017.1318744

[R13] Sinha A, & Das A. (2019). Livelihood Vulnerability of Fishery-Based Communities in Context of Climate Change: Insights From and Around Selective Fishing Grounds of South 24 Parganas, West Bengal. Journal of Geography, Environment and Earth Science International, 20, 1–12. 10.9734/jgeesi/2019/v20i130094

[R14] Steynor A, & Pasquini L. (2019). Informing Climate Services in Africa through Climate Change Risk Perceptions. Climate Services, 15, Article No.: 100112. 10.1016/j.cliser.2019.100112

[R15] Ujunwa A. (2019). Armed Conflict and Food Security in West Africa: Socioeconomic Perspective. International Journal of Social Economics, 46, 182–198. 10.1108/IJSE-11-2017-0538

[R16] van der Keur P, van Bers C, Henriksen HJ, Nibanupudi HK, Yadav S, Wijaya R, Subiyono A, Mukerjee N, Hausmann H-J, Hare M, van Scheltinga CT, Pearn G, & Jaspers F. (2016). Identification and Analysis of Uncertainty in Disaster Risk Reduction and Climate Change Adaptation in South and Southeast Asia. International Journal of Disaster Risk Reduction, 16, 208–214. 10.1016/j.ijdrr.2016.03.002

[R17] Yadav SS, & Lal R. (2018). Vulnerability of Women to Climate Change in Arid and Semi-Arid Regions: The Case of India and South Asia. Journal of Arid Environments, 149, 4–17. 10.1016/j.jaridenv.2017.08.001

